# A decade of conditional cash transfer programs for reproductive health in India: *How did equality fare*?

**DOI:** 10.1186/s12889-022-12563-9

**Published:** 2022-02-25

**Authors:** Deepali Godha, David R. Hotchkiss

**Affiliations:** 1 Independent Research Consultant, 16/1 South Tukoganj, 201 Sukh Sheetal II, Indore, MP 452001 India; 2 Department of International Health and Sustainable Development, Tulane University, School of Public Health and Tropical Medicine, New Orleans, USA

**Keywords:** Maternal health care, Inequality analysis, Conditional cash transfer programs, Janani Suraksha Yojana, Inequality, Erreygers index, Wagstaff index

## Abstract

**Background:**

Since 2005, India has implemented conditional cash transfer [CCT] programs to promote the uptake of institutional delivery services [ID]. The study aims to assess changes in wealth-based inequality in the use of ID and other maternal health care services during the first decade of Janani Suraksha Yojana and related CCT programs.

**Methods:**

Data from two Demographic and Health Surveys were used to calculate changes in service inequality from 2005 to 2015–16 in the use of three or more antenatal care [ANC] visits, ID, and postnatal care [PNC]. The changes were assessed at the national level, within high and low performing states [HPS and LPS, respectively] and within urban and rural areas of each state category. Erreygers Index [EI] and Wagstaff Index [WI], superior to concentration index, were used to gain different insights into the nature of inequality. EI is an objective measure of inequality irrespective of prevalence while WI is a combined measure of inequality and the average distribution of an indicator that puts more weight on the poor.

**Results:**

The results suggest that wealth-based inequalities decreased significantly at the national level. For ID, both indices showed a decline in both HPS and LPS though the change in WI in HPS was insignificant. For ANC, there was a significant decrease in inequality using both indices in HPS but not in LPS. For PNC, there was a significant decrease in inequality using both indices in HPS, and when using WI in LPS, but not when using EI in LPS.

**Conclusion:**

Overall, the first decade of India’s CCT programs saw an impressive reduction in EI for ID but less so for WI suggesting that the benefit of CCTs did not go disproportionately to the poor, which suggests that there is a need to reduce or eliminate the evident leakages. The improvement in uptake and inequality in ANC and PNC was not at par with ID, stressing the need to place greater focus on the continuum of care. The urban rural difference in HPS versus LPS in the changes in inequality reveals that infrastructure is important for CCTs to be more effective.

**Supplementary Information:**

The online version contains supplementary material available at 10.1186/s12889-022-12563-9.

## Background

The year 2005 was a turning point for maternal and reproductive health in India because it marked the start of a trend of using conditional cash transfer programs (CCT) as a strategy to promote institutional delivery. Janani Suraksha Yojana (JSY), which is to date the largest CCT program in the world, was launched that year in order to promote the use of institutional delivery services, and in turn, reduce maternal and neonatal mortality rates. In the decade since its launch, more than 4.55 million women [1] have been JSY beneficiaries (2015–16) with program expenditures estimated at 306.1 million USD^1^ by the Government of India [2]. The scheme provides cash assistance to both mothers as well as community health workers known as Accredited Social Health Activists (ASHA), who track each pregnancy and provide antenatal and postnatal assistance as well as breastfeeding counselling.

JSY is designed to provide differential assistance in two types of states that were categorized based on prevailing institutional delivery rates: 1) low performing states (LPS) where the cash transfer is higher and eligible women include all pregnant women delivering in public facilities and women delivering in private facilities who have the government-issued below-poverty-line (BPL) card or those belonging to a scheduled caste or tribe (SC/ST) and 2) high performing states (HPS) where the cash transfer is lower and only eligible to BPL/SC/ST women [3]. The LPS include Uttar Pradesh, Uttaranchal, Bihar, Jharkhand, Madhya Pradesh, Chhattisgarh, Assam, Rajasthan, Orissa and Jammu and Kashmir. Over the years, the scope and incentives of the program have been gradually increased with the total package of cash assistance as of 2021 (for both the mother and ASHA) amounting to 2000 INR and 1300 INR in rural and urban areas of LPS and 1400 INR and 1000 INR in rural and urban areas of HPS, respectively. The ASHA package has been divided into two equal portions – one for the antenatal care component and the other for the institutional delivery component [3].

Apart from JSY, which is a 100% centrally sponsored scheme, a slew of state specific schemes was also rolled out soon after the launch of JSY, either to improve coverage or achieve gains over and above those of JSY. The state-specific schemes for safe motherhood utilized varying combinations of expanded eligibility criteria and service packages and/or changed the amount of cash assistance and its disbursement in order to achieve further improvements beyond those of JSY. These complementary schemes included Chiranjeevi Yojana in Gujarat, Saubhagyawati Scheme in Uttar Pradesh, Janani Sahyogi Yojana in Madhya Pradesh, Janani Suvidha Yojana in Haryana, Ayushmati Scheme in West Bengal, Chiranjeevi Yojana in Assam, JSY in Orissa, and Mamta Friendly Hospital Scheme in Delhi [4–7].

It should be noted that budgetary allocations for JSY started in April 2006 while uptake took off in 2007 [8]. The rollout of the state-specific schemes differed across states but the earliest was of Chiranjeevi Yojana in Gujarat in November 2005 as a pilot in five districts. Though several schemes have been added to the maternal health portfolio over the years, such as Janani Shishu Suraksha Karyakaram (JSSK) [9], Dakshata [10], Pradhan Mantri Surakshit Matritva Abhiyan [11], and LaQshya [12], these are not conditional cash transfer schemes and JSY remains the main one.

Over the years, issues regarding the implantation and impact of JSY have received a significant attention from researchers. The earliest studies were mainly conducted to understand process and implementation gaps but were limited geographically [13–19]. Later, national-level evaluations of the impact on maternal and neonatal health outcomes using advanced econometric techniques were carried out by Lim et al. [20], Powell-Jackson et al. [21], Joshi and Sivaram [22], and Carvalho & Rokicki [23]. Overall, the studies found evidence of a positive impact of JSY on the uptake of institutional delivery and to a smaller extent on antenatal care and skilled birth attendance. The studies also found substantial variation in the effect of JSY on institutional delivery and skilled birth assistance across states and found that the impacts were higher among high-focus states (or LPS) compared to non-focus states (or HPS). Lim et al. [20] and Carvalho & Rokicki [23] also found evidence of differential targeting by age, educational attainment, and the caste of women beneficiaries and that the receipt of cash assistance was not highest for the poorest and least educated. Powell-Jackson et al. [21] and Joshi & Sivaram [22] found that the increased use of maternity services associated with JSY was higher among poorer and less educated women, in rural areas as compared to urban areas, and in LPS as compared to HPS. There have been other previous assessments that were less rigorous or restricted geographically [3, 24–26].

However, formal inequality analysis focusing on JSY has so far been done in only a few studies, though with increasing technical rigor. The first such study, conducted by Modugu et al. [27], looked at variations in the percentage of JSY use at the state level and by socio-demographic characteristics in India. Using an ecological study design in nine low performing states, Randive et al. [28] analyzed concentration curves and indices along with their decomposition to assess inequality in institutional delivery uptake and access to emergency obstetric care after the introduction of JSY. The study used area-level socioeconomic measures and different surveys ranging from DLHS-3 (2007–08), Annual Health Survey (2010–11 and 2012), and Census of India (2011). The results suggested that there was reduced inequality in institutional delivery (from 0.19 in 2004–06 to 0.09 in 2010) and increased inequality in maternal mortality post-JSY. Joe et al. used the Morbidity and Health Care Survey and the Social Consumption Health Survey conducted by the National Sample Survey Office in 2004 and 2014 respectively to analyze the concentration index (CI) to quantify public sector’s contribution in improving equity in use of institutional delivery at the national level [29]. In the period 2004 to 2014, the authors found a reduction in CI for institutional deliveries at the national level (from .239 to 0.054), in the public sector (from 0.086 to - 0.082), and in home deliveries (− 0.182 to − 0.256) but less so in the private sector (0.383 to 0.300). In a study based on data from high-focus states, Vellakkal et al. analyzed relative indices of inequality and pre-post difference-in-difference to show that inequity in institutional deliveries reduced slower in the 2007–08 period but had a steeper decline in the 2011–12 period [30]. The latter was true for antenatal care as well [30]. Thongkong et al. [31] used a corrected concentration index (CCI also known as Erreygers Index) and its decomposition to find evidence of pro-rich inequalities in institutional deliveries and receipt of JSY benefit in five districts of Jharkhand and Odisha states in the period 2009–10. The study estimated CCI of 0.16 in Odisha and 0.30 in Jharkhand for institutional deliveries and 0.10 in Odisha and 0.18 in Jharkhand for JSY receipt. Recently, Mishra et al. conducted a geospatial analysis to look at regional disparity in coverage of JSY scheme among Indian women with institutional deliveries over India districts [32]. The authors used Local Indicator for Spatial Association (LISA) maps to show that JSY coverage was clustered in districts of a few states only while the spatial error models depicted an increase in the benefit of JSY associated with lack of education and poverty.

Although previous studies have found that JSY has had a significant impact on the uptake of institutional deliveries in India, the question of how JSY along with the complementary CCTs have influenced inequality in the use of institutional delivery services and other types of maternal healthcare services at the national level has not been fully investigated. Previous studies have been mainly confined to the immediate period after the launch of the JSY scheme or have been geographically limited. The one national study that has been carried out, by Joe et al., that had a lengthy period of 2004–2014, analysed inequality using concentration indices, but previous researchers have demonstrated that such indices are not well-suited for binary variables. Though Thongkong et al. [31] have used EI, an index more suited to binary variables, the study was confined to the 2009–2010 period and that too in five districts of Odisha and Jharkhand.

The study presented in this article fills these gaps by investigating changes in service inequality that occurred over a longer period of analysis (a decade as opposed to much more limited time periods used in several studies); is nation-wide as opposed to the sub-national scope of most of the previous studies on this topic; uses two much superior inequality indices, EI and WI, that offer differing insights into the inequality impact of CCTs as opposed to most of the other studies that have used CI which is not well suited to binary variables or one study that has used only EI; and analyzes three maternal healthcare indicators that provide a comprehensive view on the maternal health care continuum. Apart from service inequality, the study also analyses participation in the CCTs. The data used for the study comes from population-based household survey data from the 2005 and 2015–16 Demographic and Health Surveys, the latest of which was not available to the researchers of the studies cited above.

## Data

The study utilizes two India Demographic and Health Surveys (DHS) conducted in 2005–06 (NFHS-III [33]) and 2015–16 (NFHS-IV [34]). The two district-level health surveys (DLHS)^2^ conducted during this period have not been considered because of comparability issues [20, 23]. In the case of NFHS III, the data analysis was restricted to the year 2005 and earlier by including only those surveyed before January 2006. This was done to demarcate a clear period before the roll-out of the CCTs.

Indicators of service delivery were selected to assess changes in the uptake of antenatal, natal, and postnatal maternal health services. The indicators included in this study were: three or more antenatal (ANC) visits; institutional delivery (ID); and postnatal care to the mother within 2 days of delivery by a health provider (PNC). Though analysis was also conducted for skilled attendance at birth, the results have not been presented because they were similar to those of ID. For each indicator, the analysis was restricted to the most recent delivery in the past 5 years. Women beneficiaries were defined as those who received cash assistance for delivery either under JSY or one of the related state-specific government schemes. The final sample included 34,036 women from NFHS-III and 189,143 women from NFHS-IV.

## Methods

Research on improving inequality measurement has been an ongoing process. While the widely used CI requires that the health variable be ratio-scaled without an upper bound, researchers found that it had issues with ranking binary variables with bounds. This led to the derivation of the Erreygers Index (EI) and the Wagstaff Index (WI) [35]. The equations used to estimate the indices can be found in the end-note.^3^

The two indices differ in terms of how the most unequal society is defined [37]. Under the WI, it is assumed that the most unequal society occurs when only the richest share of individuals utilizes the health care service, and this share is equal to the observed health care utilization rate. In addition, WI gives more weight to the extremes. On the other hand, under the EI, the calculation of inequality does not involve the observed utilization rate, and as a result, is independent of the rate. This means that the values of EI and WI only coincide when the utilization rate is equal to 50%, and that the difference between the values of EI and WI becomes greater as the utilization rate becomes larger than 50%.

There is an ongoing debate on the superiority of one index over the other, but for objective indicators, the two indices provide insight from different perspectives because of the differing value judgements that are the basis of indices. EI provides an absolute value judgement and is independent of the prevalence of the indicator while WI provides an overall measure of achievement that captures the extent of inequality of an indicator and its average distribution [36]. WI takes into account the initial prevalence and inequality level and weighs the inequality higher if the prevalence lies at extreme ends, providing insight from a policy perspective where the aim is that improvements disproportionately benefit the poor [36]. Such perspective becomes important when the context is specifically concerned with health of the poor given that maternal mortality is more prevalent among the uneducated and the poor who cannot afford the services [37]. For either index, a positive value indicates a pro-rich concentration, a negative value indicates pro-poor concentration while a value of 0 indicates no inequality.

Since the indicators used in this study are all objective but based on different value judgements, we refrain from choosing one index over the other and instead use the difference in their value judgements to gain a deeper understanding of the inequality context. A critique of earlier evaluations of JSY studies was the misclassification of beneficiaries due to blurred lines between the central scheme of JSY and the various state schemes. We have ignored these distinctions to examine the pre- and post-inequality scenario that concerns all CCTs aimed at improving demand of maternal health services. Even so, we believe that the results are primarily a reflection of JSY, given its scale (88.9% of the CCT beneficiaries received benefits from JSY) and that the state schemes are mainly an adaptation along similar lines. So, we adhere to the different geographical categories devised for JSY cash transfers. Accordingly, the indices have been estimated separately for the total country and for the two state categories, HPS and LPS, which were further subdivided into rural and urban areas. In addition to the indices, the prevalence of each indicator along with its distribution by household wealth quintiles in HPS and LPS and their urban and rural areas was also estimated. For the latter, weighted quintiles were created separately for urban and rural areas. The study also investigates participation in the CCT programs.

All analyses were generated using the ‘conindex’ command in Stata 15.1 [38] using survey weights. Results, in the form of prevalence estimates and the indices (EI and WI) for each indicator, are presented as figures. All three parameters are presented for the national level and for each state category (HPS and LPS). Results by urban/rural area of residence for each of the two state categories are available as Supplementary tables (See Supplementary Tables 1 and 2). In addition, two statistical tests were carried out to test if the differences were significant across survey years for EI and WI. We used the z-statistic for these tests which assumes large sample sizes. The distribution of the indicators by wealth quintiles and by area of residence within each state category are illustrated through another series of figures.

## Results

**Fig. 1:**
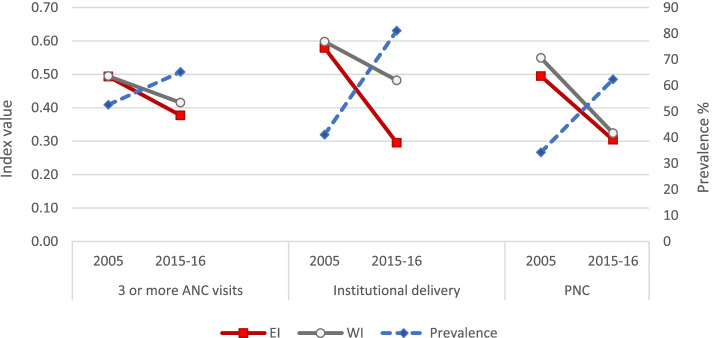
Prevalence and inequality indices of maternal health care indicators at the national level, by survey year

The pre-post analysis at the national level revealed that the prevalence of having had three or more ANC visits increased by around 13 percentage points over the study period while ID and PNC almost doubled (Fig. 1). Inequality, as measured by both EI and WI, decreased for each of the service indicators.

### Three or more ANC visits

**Fig. 2:**
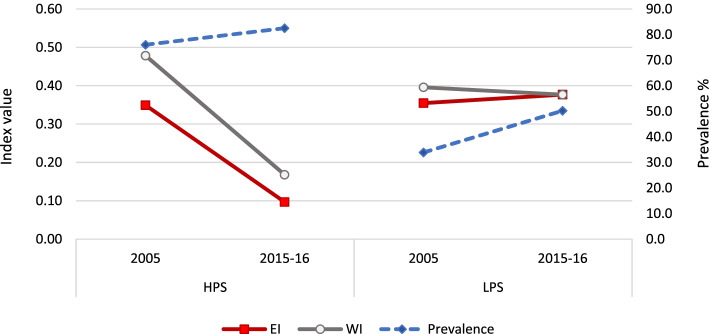
Prevalence and inequality indices of ANC visits, by survey year and state category

Figure 2 shows that the prevalence of having had three or more ANC visits increased (in absolute terms) more in LPS (from 33.9% in 2005 to 50.2% in 2015–16) as compared to HPS (from 76.0% in 2005 to 82.5% in 2015–16). In the case of HPS, the rise in rural areas (12.8 percentage points) was negated by a decline in the urban areas (4.4 percentage points). In LPS, the prevalence rose in both urban and rural areas but was much higher in rural areas (17.3 percentage points in rural areas versus 11.3 percentage points in urban areas). However, this did not translate into a corresponding change in inequality. Despite an increased prevalence of 16.3 percentage points in LPS, there was no significant change in EI, whereas it dropped to almost one-fourth in HPS by .28 points. The EI drop in HPS was due to drops in both urban and rural areas in spite of a drop in prevalence in the urban areas of HPS.

An almost similar picture was observed for WI, as it dropped to one-third in HPS but showed no significant change in LPS. In HPS, the drop was more in urban areas as compared to rural areas, but in LPS, the drop in WI in urban areas was offset by its rise in rural areas.

**Fig. 3:**
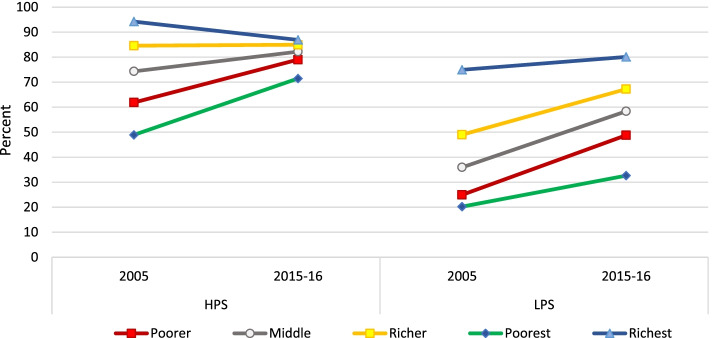
Distribution of three or more ANC visits across wealth quintiles, by survey year and state category

Figure 3 shows the distribution of having had three or more ANC visits by survey years across wealth quintiles in the two state categories and their urban and rural areas. In HPS, the prevalence of three of more ANC visits across survey years changed more among the lower two wealth quintiles while it decreased in the richest wealth quintile. In the urban areas of HPS, this change was in the negative- while prevalence across survey years remained almost same among the poorest, it dropped among the richest. In the rural areas of HPS, the change was not much in the top two wealth quintiles whereas it was 15.7 and 18.6 percentage points among the lowest two wealth quintiles. In LPS, a high uptake was observed among the middle three wealth quintiles. In urban areas of LPS, the change was positive in the lower three wealth quintiles with a zero and negative change in the top two wealth quintiles respectively. In rural LPS, the increase in inequality that occurred despite the increase in prevalence is explained by an almost uniform increase in prevalence across the wealth quintiles with the poorest showing the lowest increase.

### Institutional delivery

**Fig. 4:**
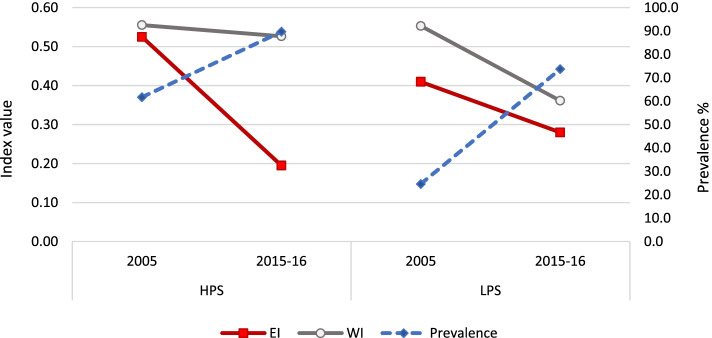
Prevalence and inequality indices of institutional delivery, by survey year and state category

Figure 4 shows that the prevalence of utilized ID services increased almost 1.5 times in HPS and almost 3 times in LPS from 2005 to 2015–16. In HPS, the increase was mainly in rural areas by 36.6 percentage points. In LPS, the increase was observed in both urban and rural areas by 31.6 and 53 percentage points respectively. Even though the relative and absolute increase in prevalence was lower in HPS as compared to LPS, the corresponding impact on EI was higher in HPS. In HPS, the fall in EI to almost one-third was contributed by a drop of .18 points in urban area (to almost one-third) and of .23 points in rural area (to half). In LPS, the drop in EI was entirely contributed by the drop in urban areas of .36 points with no significant change in rural areas. On the other hand, the change in WI was insignificant in HPS while it fell to one-third in LPS. The drop in WI was relatively more in urban areas of LPS (by .19 points) as compared to rural areas (by .10 points).

**Fig. 5:**
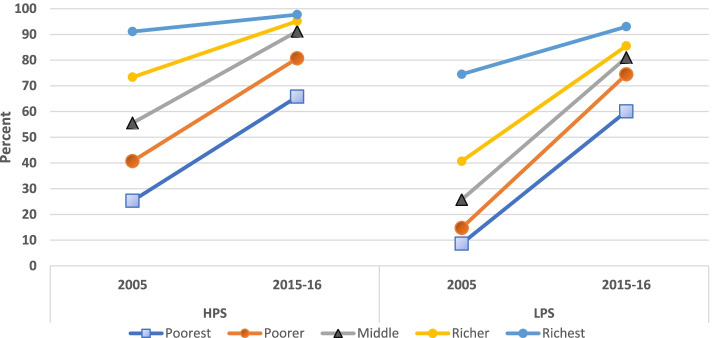
Distribution of institutional delivery across wealth quintiles, by survey year and state category

Figure 5 shows that the increased coverage of ID in HPS was the highest among the poorest and this difference reduced with increasing wealth. A similar picture was observed in urban areas of HPS with an increase of 17.3 percentage points among the poorest as compared to 1.3 percentage points among the richest. Comparatively, the uptake was relatively higher across all the wealth quintiles in the rural areas of HPS, though it showed the same gradient decline from poorest to richest (an increase of 37.6 percentage points among the poorest as compared to 14 percentage points among the richest). In LPS, the increase in prevalence was more than 50 percentage points in lower 3 wealth quintiles. The urban areas of LPS showed a marked difference in uptake across wealth quintiles- of around 44 percentage points among the poorest as compared to 2 percentage points among the richest. In rural areas of LPS, the increase was more uniform in the lower four wealth quintiles of around 47–53 percentage points.

### Postnatal care within two days of delivery by a health provider

**Fig. 6:**
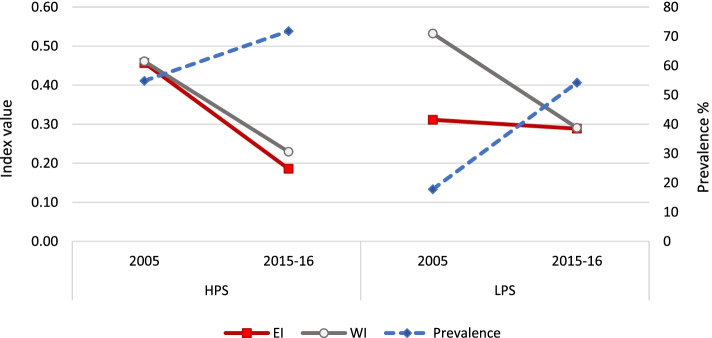
Prevalence and inequality indices of PNC, by survey year and state category

Figure 6 shows that the prevalence of having had PNC increased more in LPS (from 17.8% in 2005 to 54.2% in 2015–16) as compared to HPS (from 54.8% in 2005 to 71.8% in 2015–16). In case of HPS, the rise in rural areas (23.7 percentage points) was almost five times that of urban areas (4.9 percentage points). In LPS, the prevalence rose in both urban and rural areas but the difference over time was much higher in rural areas (38.9 percentage points in rural areas versus 25.1 percentage points in urban areas). However, this did not translate into a corresponding change in inequality. Despite an increased prevalence of 36.4 percentage points in LPS there was no significant change in EI, whereas it dropped to two-fifths in HPS by .27 points. The EI drop in HPS was similar in both urban and rural areas.

An almost similar picture was observed for WI – which dropped to around half in both HPS and LPS by around .23 points. In both HPS and LPS, the drop was larger in urban areas (.20 and .22 points respectively) than in rural areas (.15 and .14 points respectively).

**Fig. 7:**
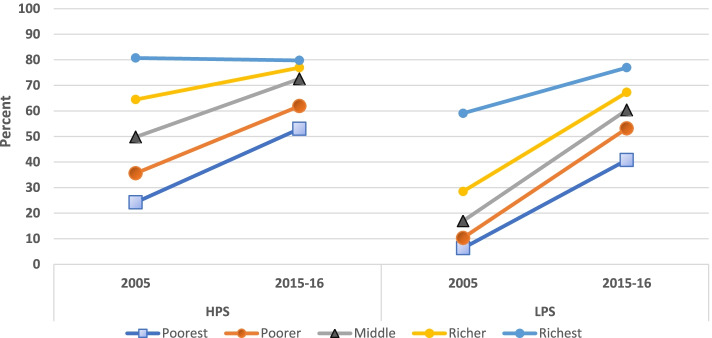
Distribution of PNC across wealth quintiles, by survey year and state category

Figure 7 shows the distribution of PNC by survey years across wealth quintiles in the two state categories and their urban and rural areas. In HPS, the prevalence of PNC across survey years changed more among the lower three wealth quintiles while in LPS, it decreased in all wealth quintiles except the topmost. In urban areas of HPS, this change was small (8.6 to 2.9 percentage points in lower three wealth quintiles). In the rural areas of HPS, the change was not much in the topmost wealth quintile whereas it was 27.5 and 22.3 percentage points in the lowest two wealth quintiles. In LPS, a high uptake was observed among all wealth quintiles. In urban areas of LPS, the change was of 30.7 percentage points in the lowest wealth quintile followed by 24.3 percentage points in the second lowest wealth quintile. In rural LPS, the increase in inequality despite the increase in prevalence is explained by an almost uniform increase in prevalence across the wealth quintiles.

### Participation in CCT programs among women utilizing maternal health care

In addition to assessing inequality in service utilization, we also investigated the extent to which women in 2015–16, who used maternal health care services, reported that they participated in the CCT programs in order to gain insights on the differences in program participation between women in HPS and LPS and how well the programs were targeted to the poor. Among the full sample of women, almost one-third of maternal health care users reported that they were CCT beneficiaries, 69.6% of which were in LPS and 30.4% of which were in HPS) (not shown).

**Fig. 8:**
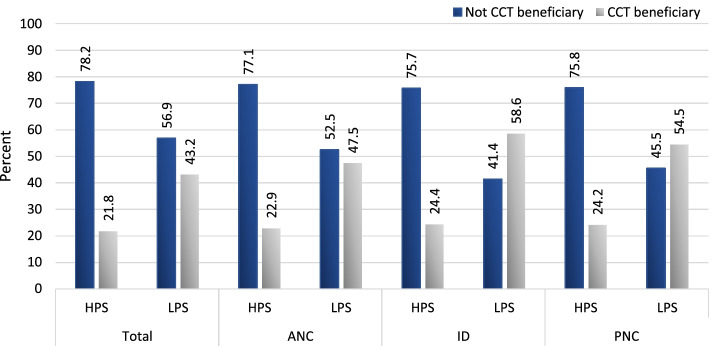
Percent distribution of service users by whether they received CCT benefits, by type of service and state category

Figure 8 shows a percentage distribution of service users by whether they reported that they were CCT beneficiaries, by type of service and state category. As Fig. 8 shows, the percentages of service users of ANC, institutional delivery, and postnatal care who were CCT beneficiaries were substantially higher within LPS than within HPS. For example, 47.5% of ANC service users, 58.6% of ID service users, and 54.5% of PNC service users in LPS were CCT beneficiaries, compared to 22.9% of ANC service users, 24.4% of ID service users, and 24.2% of PNC service users in HPS.

Table 1 extends the analysis of CCT program participation by disaggregating women within each wealth quintile by type of service, state category and area of residence. Within LPS, the percentage of urban service users who were CCT beneficiaries decreased with increasing wealth, while the comparable percentages among rural service users was more uniform across the wealth quintiles. Within HPS, similar urban/rural patterns emerged, although the percentage of HPS service users who participated in CCT programs was substantially lower than the percentages of LPS service users for each area-specific and wealth-group specific category.

**Table 1:** Percent of service users that were CCT beneficiaries, by state category, type of service, area of residence, and wealth quintile

State category, wealth quintile	Three or more ANC visits		Institutional delivery		Postnatal care	
	Urban	Rural	Urban	Rural	Urban	Rural
**HPS**						
Poorest	17.6	19.1	20.8	28.6	19.9	25.1
Poor	21.3	21.5	23.1	28.8	22.6	27.7
Middle	18.6	25.1	19.5	30.0	19.8	28.7
Rich	11.6	26.3	12.1	28.8	13.1	27.9
Richest	7.0	19.4	7.6	19.8	7.8	19.7
**LPS**						
Poorest	41.5	40.5	54.9	66.1	51.5	62.0
Poor	39.5	46.8	47.1	65.2	44.5	61.3
Middle	34.5	50.8	39.7	63.2	36.2	60.6
Rich	25.4	49.0	29.4	57.7	27.3	55.5
Richest	17.3	39.7	19.5	45.2	17.7	42.6

## Discussion

The purpose of the study was to assess changes in wealth-based inequality in the use of ID and other maternal health care services during the first decade of India’s JSY and related CCT programs, and to get nuanced insights into the context through the use of two indices that are well suited for assessing binary indicators of service use, EI and WI.

Overall, the national-level results suggest that not only did the utilization of maternal health care service increase substantially over the 2005 to 2015–16 study period, but also that wealth-based inequalities in the use of services decreased significantly. Similar results were observed when the analysis was disaggregated by type of state (HPS and LPS). For ID, though both indices of inequality showed a decline in both HPS and LPS, the change in WI in HPS was insignificant. For ANC, there was a significant decrease in inequality using both EI and WI in HPS but not in LPS. For PNC, there was a significant decrease in inequality using both EI and WI in HPS, and when using WI in LPS, but not when using EI in LPS.

Because the design of the CCTs was different in urban and rural areas, separate analyses of women in urban and rural areas were carried out. Based on the magnitude and the direction of changes in the two indices over time at the national level and by state category and area if residence, six different scenarios were identified (Table 2).

**Table 2:** Different scenarios based on change in indices- EI and WI, over time

Scenarios	National	HPS			LPS		
		Total	Urban	Rural	Total	Urban	Rural
1. EI decreased more than WI	ID					ID	
2. WI decreased more than EI			Three or more ANC visits				
3. Decrease almost similar^a^	Three or more ANC visits, PNC	Three or more ANC visits, PNC	PNC	Three or more ANC visits, PNC	ID	Three or more ANC visits, PNC	
4. Both increased/changed insignificantly					Three or more ANC visits (EI NS, WI NS)		Three or more ANC visits (WI ▲, EI ▲)
5. EI decreased but WI did not/small reduction/increased		ID (EI ▼, WI NS)	ID (EI ▼, WI NS)	ID (EI ▼, WI NS)			
6. WI decreased while EI did not/small reduction/increased					PNC (WI ▼, EI NS)		ID (WI ▼, EI NS) PNC (WI ▼, EI ▲)

^a^Absolute difference between difference in indices over time < =.06 - No statistical tests done

▲- Increased; ▼- Decreased; *NS* Not significant

The scenario where EI declined more than WI indicates that the poor did not benefit disproportionately. This occurred for ID at the national level and in urban LPS. Given that prevalence in ID increased markedly, policymakers should next aim to ascertain that further service uptake is concentrated amongst the poor.

Situations in which WI dropped more than EI indicate that improvement in service uptake was “disproportionately concentrated amongst the poor” but the absolute improvement was not necessarily greater. This may not necessarily be a desirable outcome for policy makers, as there was a decline over time in the uptake of three or more ANC services in urban HPS. While there was no change in prevalence amongst the poorest quintile, the prevalence declined in all other wealth quintiles. Further research is needed to understand why the uptake of services declined.

A change in EI and WI of the same magnitude and direction means that the increase in service uptake was greater for the poor groups as compared to the richer groups in absolute quantity as well as proportions, and as a result, the relative differences between the utilization rates of the poor and richer groups decreased. This change was pro-poor and was observed for three or more ANC visits at the national level, in rural HPS and urban LPS; and PNC at the national level, in urban and rural HPS, and urban LPS.

Scenario 4, in which both indices showed an increase or the change was insignificant, is an indication that mainly the rich benefitted. The change was insignificant for three or more ANC visits in LPS. Both indices increased in the case of three or more ANC visits in rural LPS, despite an increase in prevalence and a heftier cash transfer that was available to women in rural areas.

Scenarios in which EI declined but not WI (or the change was insignificant) reflect a greater incremental change in service uptake amongst the poorest quintile, but the distribution of additional uptake is not concentrated amongst the poor. This happened for ID in HPS (total, urban, and rural).

In the cases where WI declined but EI did not, or the change was insignificant, even though there was not much incremental change, the analysis does suggest that the changes that did occur went disproportionately to the poor. This was the case for ID in rural LPS and PNC in LPS (total and rural).

Scenario 4 suggests an area this is in need of greater prioritization for policy makers – improving uptake of three or more ANC services in LPS. Though the uptake of ANC services might have increased in the earlier period as suggested by Lim et al. [20], the results after the first decade since JSY and related CCTs were first implemented show that the magnitude of improvement in ANC and PNC services was not at par with those observed in ID services. This was foreseen by previous researchers [17] who stressed the need to focus more on the continuum of care, and not just ID services. Starting in 2009–10, design changes that linked cash-transfers to antenatal care were gradually implemented, but these were only for ASHAs. However, these must include PNC services too and design features should also focus on mothers in order to improve the current imbalance [31].

Even though the cash transfer was higher in LPS as compared to HPS, the drop in EI was higher for all indicators in HPS as compared to LPS, while the drop in WI was higher for ID in LPS as compared to HPS. Further differentiation by area of residence showed that the drop in EI was higher in rural areas as compared to urban areas of HPS while the opposite was true for the drop in WI (higher in urban areas as compared to rural areas) except for PNC. In the case of LPS, the drops in EI and WI were higher in urban areas as compared to rural areas. This indicates that focusing only cash transfers is insufficient to improve uptake of maternal health services. The urban-rural difference in HPS versus LPS in changes in inequality over the study period reveals that cash transfer plays a bigger role, if and only if, the appropriate infrastructure is in place.

The analysis of CCT program participation based on the 2015–16 DHS sample indicates that the large scale and intensive implementation of CCT schemes resulted in almost one-third of all maternal health care users at the national level participating in the CCT programs. Two-thirds of these program beneficiaries were in LPS while one-third were in HPS. The analysis of program participation by wealth quantiles suggests that program leakage to the richer population is evident, particularly within rural areas in both state categories. This indicates that improvements in targeting are needed, and this could potentially yield further progress in reducing wealth-based service inequality.

The study has certain limitations and in no way do our results on changes in wealth-based service inequality provide evidence of causality. The results cannot be fully attributed to the CCT programs as there are other factors that might have played important roles, including improvements in the economy, infrastructure, and awareness. In addition, CCT beneficiaries comprised only one-third of the study population. However, since the CCTs did provide the necessary motivation to those who otherwise would not have used the maternal health care services and were aimed at the poor, it is perfectly reasonable to look at how inequality fared. The scope of the scheme may in itself have aided in improving the awareness, attitudes, and behavior of the non-beneficiaries as well as the beneficiaries.

## Conclusion

Overall, the results suggest that inequality in the use of maternal health care services decreased during the first decade of India’s JSY program. That wealth-based inequalities declined in both HPS and LPS, despite the differences in how the program was targeted, suggests that both targeting based on means testing (which assesses whether the household qualifies as being impoverished under the definition of the program) as well as geographic targeting can yield improvements in the equality of maternal health care delivery.

The use of both inequality measures (EI vs. WI) provides further insights into the progress made towards improvement in maternal health, particularly among the poor. It has been acknowledged that achieving a decline in EI is much more difficult than a reduction in WI [39]. Also true is that improvement in prevalence does not equate to a disproportionate benefit to the poor [40]. Though the marked reduction in EI for ID at the national level and in urban LPS is commendable, the reduction was less so for WI suggesting that the benefit of CCTs did not go disproportionately to the poor. Measures to reduce or eliminate the evident leakage of CCTs to the non-poor (which was higher in rural areas) may help achieve this policy aim.

The improvement in the uptake and inequality in ANC and PNC was not at par with ID, stressing the need to place greater focus on the continuum of care. The urban-rural difference in HPS versus LPS in the change of inequality reveals that health infrastructure (facilities, trained health personnel, diagnostics, medicines, and equipment) is important for CCTs to be more effective [41].

While assessing the role of the design of the JSY program on wealth-based service inequality is beyond the scope of the study, the results suggest the need to strengthen efforts to minimize program leakages, improve the continuum of care, and improve infrastructure in order to yield a greater synergetic effect of the CCTs on service equality.

## Supplementary Information

**Additional file 1.**

## Data Availability

The datasets used for the analyses of this article are publicly available in The DHS Program repository. https://dhsprogram.com/methodology/survey/survey-display-264.cfm [33] and https://dhsprogram.com/data/dataset/India_Standard-DHS_2015.cfm?flag=0 [34].

## Abbreviations

ANC: Antenatal Care
ASHA: Accredited Social Health Activists
BPL: Below Poverty Line
CCI: Corrected Concentration Index
CCT: Conditional Cash Transfer Programs
CI: Concentration Index
DHS: Demographic and Health Surveys
EI: Erreygers Index
HPS: High Performing States
ID: Institutional Delivery
JSY: Janani Suraksha Yojana
LPS: Low Performing States
NFHS: National Family Health Survey
PNC: Postnatal Care
SC: Scheduled Caste
ST: Scheduled Tribe
WI: Wagstaff Index

## Footnotes

1 As per average exchange rate in *2016.*https://www.exchangerates.org.uk/USD-INR-spot-exchange-rates-history-2016.html*. Accessed 28 Oct 2021.*

2 The DLHS are household surveys conducted by the Government of India to gather district-level information on utilization and people’s perception of quality of maternal and child health services and other reproductive health services by public health facilities (Source: http://rchiips.org).

3 $E=\frac{8}{n^2}{\sum}_{i=1}^n{z}_i{h}_i$; $W=\frac{2}{n^2\left(1-{\mu}_h\right){\mu}_h}{\sum}_{i=1}^n{z}_i{h}_i$ where n is the number of individuals in a given population; ${z}_i=\frac{\left(\mathrm{n}+1\right)\ }{2}-{\uplambda}_i$ (where λ_*i*_ is the socioeconomic rank of the individual ranging from the richest (λ_*i*_ =1) to the poorest (λ_*i*_ =n)); *z*_*i*_ is the health variable; and *μ*_*h*_ is the mean of the health variable.
