# Supplementary material for: A decade of conditional cash transfer programs for reproductive health in India: How did equality fare?
Source: BMC Public Health. 2022 Feb 25;22:394. doi: 10.1186/s12889-022-12563-9 (PMC8876831; doi:10.1186/s12889-022-12563-9)
Supplement: Supplementary file 1 — Additional file 1. [file 12889_2022_12563_MOESM1_ESM.docx]

Supplementary Table 1. Index values for each indicator by survey year, state category, and area.

|  | **Erreygers Index** | | | **Wagstaff Index** | | |  |
| --- | --- | --- | --- | --- | --- | --- | --- |
| **Type of service** | **2005** | **2015-16** | **z-statistic^@^** | **2005** | **2015-16** | **z-statistic^@^** |  |
| **Three or more ANC visits** |  |  |  |  |  |  |  |
| **Total** |  |  |  |  |  |  |  |
| National | 0.49 | 0.38 | *** | 0.49 | 0.42 | *** |  |
| HPS | 0.35 | 0.10 | *** | 0.48 | 0.17 | *** |  |
| LPS | 0.35 | 0.38 | NS | 0.40 | 0.38 | NS |  |
| **HPS** |  |  |  |  |  |  |  |
| Urban | 0.19 | 0.08 | *** | 0.45 | 0.15 | *** |  |
| Rural | 0.33 | 0.11 | *** | 0.38 | 0.18 | *** |  |
| **LPS** |  |  |  |  |  |  |  |
| Urban | 0.51 | 0.35 | *** | 0.51 | 0.39 | *** |  |
| Rural | 0.24 | 0.32 | *** | 0.29 | 0.32 | * |  |
|  |  |  |  |  |  |  |  |
| **Institutional Delivery** |  |  |  |  |  |  |  |
| **Total** |  |  |  |  |  |  |  |
| National | 0.58 | 0.30 | *** | 0.60 | 0.48 | *** |  |
| HPS | 0.52 | 0.20 | *** | 0.56 | 0.53 | NS |  |
| LPS | 0.41 | 0.28 | *** | 0.55 | 0.36 | *** |  |
| **HPS** |  |  |  |  |  |  |  |
| Urban | 0.29 | 0.11 | *** | 0.49 | 0.48 | NS |  |
| Rural | 0.45 | 0.22 | *** | 0.45 | 0.48 | NS |  |
| **LPS** |  |  |  |  |  |  |  |
| Urban | 0.58 | 0.23 | *** | 0.59 | 0.41 | *** |  |
| Rural | 0.26 | 0.27 | NS | 0.43 | 0.33 | *** |  |
|  |  |  |  |  |  |  |  |
| **Postnatal Care** |  |  |  |  |  |  |  |
| **Total** |  |  |  |  |  |  |  |
| National | 0.49 | 0.30 | *** | 0.55 | 0.32 | *** |  |
| HPS | 0.46 | 0.19 | *** | 0.46 | 0.23 | *** |  |
| LPS | 0.31 | 0.29 | NS | 0.53 | 0.29 | *** |  |
| **HPS** |  |  |  |  |  |  |  |
| Urban | 0.29 | 0.11 | *** | 0.35 | 0.15 | *** |  |
| Rural | 0.40 | 0.21 | *** | 0.40 | 0.25 | *** |  |
| **LPS** |  |  |  |  |  |  |  |
| Urban | 0.50 | 0.27 | *** | 0.52 | 0.29 | *** |  |
| Rural | 0.18 | 0.26 | *** | 0.40 | 0.26 | *** |  |
| **^@^** Assuming large sample  Note: “NS” indicates the z-statistic is not statistically significant. | | | | | | | |

Supplementary Table 2. Prevalence (%) of each indicator by survey year, state category, and area.

|  | **2005** | **2015-16** |
| --- | --- | --- |
| **Three or more ANC visits** |  |  |
| **Total** |  |  |
| National | 52.6 | 65.2 |
| HPS | 76.0 | 82.5 |
| LPS | 33.9 | 50.2 |
| **HPS** |  |  |
| Urban | 88.2 | 83.8 |
| Rural | 68.8 | 81.6 |
| **LPS** |  |  |
| Urban | 55.4 | 66.7 |
| Rural | 28.9 | 46.2 |
|  |  |  |
| **Institutional delivery** |  |  |
| **Total** |  |  |
| National | 41.1 | 81.1 |
| HPS | 61.7 | 89.7 |
| LPS | 24.6 | 73.7 |
| **HPS** |  |  |
| Urban | 81.6 | 94.0 |
| Rural | 50.0 | 86.6 |
| **LPS** |  |  |
| Urban | 51.8 | 83.4 |
| Rural | 18.3 | 71.4 |
|  |  |  |
| **Postnatal care** |  |  |
| **Total** |  |  |
| National | 34.28 | 62.36 |
| HPS | 54.82 | 71.8 |
| LPS | 17.79 | 54.19 |
| **HPS** |  |  |
| Urban | 70.31 | 75.17 |
| Rural | 45.73 | 69.38 |
| **LPS** |  |  |
| Urban | 40.07 | 65.15 |
| Rural | 12.66 | 51.56 |
